# Inclusion of calcium phosphate does not further improve in vitro and in vivo osteogenesis in a novel, highly biocompatible, mechanically stable and 3D printable polymer

**DOI:** 10.1038/s41598-022-21013-w

**Published:** 2022-10-10

**Authors:** Nazanin Owji, Nandin Mandakhbayar, Jae-Ryung Cha, Andrew R. Padalhin, Zalike Keskin Erdogan, Alaa Aldaadaa, Taleen Shakouri, Prasad Sawadkar, Oliver Frost, Hae-Won Kim, Elena García-Gareta, Jonathan C. Knowles

**Affiliations:** 1 Division of Biomaterials and Tissue Engineering, Eastman Dental Institute, University College London, London, UK; 2 Institute of Tissue Regeneration Engineering (ITREN), Dankook University, Cheonan, Republic of Korea; 3 Department of Chemistry, Dankook University, Cheonan, Republic of Korea; 4 Department of Nanobiomedical Science & BK21 PLUS NBM Global Research Centre for Regenerative Medicine, Dankook University, Cheonan, Republic of Korea; 5 College of Medicine, Beckman Laser Institute Korea, Dankook University, Dankook, Cheonan, Chungcheongnam-do Republic of Korea; 6 UCL Eastman-Korea Dental Medicine Innovation Centre, Dankook University, Cheonan, Republic of Korea; 7 Regenerative Biomaterials Group, The RAFT Institute & The Griffin Institute, Northwick Park and Saint Mark’s Hospitals, Harrow, UK; 8 Aragonese Agency for R&D (ARAID) Foundation, Zaragoza, Aragón Spain; 9 Multiscale in Mechanical & Biological Engineering Research Group, Aragon Institute of Engineering Research (I3A), School of Engineering & Architecture, University of Zaragoza, 50018 Zaragoza, Spain

**Keywords:** Biomaterials, Cell biology, Chemical biology

## Abstract

At a time of unpredictable challenges for health, one trend is certain: there is an exceedingly high demand for functional implants, particularly bone grafts. This has encouraged the emergence of bone tissue engineering substitutes as an alternative method to conventional bone grafts. However, the current approaches in the field face several limitations that have prevented the ultimate translation into clinical settings. As a result, many attempts have been made to fabricate synthetic bone implants that can offer suitable biological and mechanical properties.

Light curable methacrylate-based polymers have ideal properties for bone repair. These materials are also suitable for 3D printing which can be applicable for restoration of both function and aesthetics. The main objective of this research was to investigate the role of calcium phosphate (CaP) incorporation in a mechanically stable, biologically functional and 3D printable polymer for the reconstruction of complex craniofacial defects. The experimental work initially involved the synthesis of (((((((((((3R,3aR,6S,6aR)- hexahydrofuro[3,2-b]furan-3,6-diyl)bis(oxy))bis(ethane-2,1- 48 diyl))bis(oxy))bis(carbonyl))bis(azanediyl))bis(3,3,5-trimethylcyclohexane-5,1- 49 diyl))bis(azanediyl))bis(carbonyl))bis(oxy))bis(ethane-2,1-diyl) bis(2-methylacrylate) referred to as CSMA and fabrication of composite discs via a Digital Light Printing (DLP) method. The flow behaviour of the polymer as a function of CaP addition, surface remineralisation potential, in vitro cell culture, using MC3T3 and Adipose-Derived Mesenchymal Stem Cells (ADSCs) and *ex ovo* angiogenic response was assessed. Finally, in vivo studies were carried out to investigate neo-bone formation at 4- and 8-weeks post-implantation. Quantitative micro-CT and histological evaluation did not show a higher rate of bone formation in CaP filled CSMA composites compared to CSMA itself. Therefore, such polymeric systems hold promising features by allowing more flexibility in designing a 3D printed scaffold targeted at the reconstruction of maxillofacial defects.

## Introduction

The worldwide incidence of bone disorders has increased steeply in the past decade^1^; in the craniofacial region specifically, congenital disorders, traumatic incidents and tumour removals are the major causes of bone loss. Victims of facial injuries can sustain severe disfigurements, with a resultant emotional and psychological impact on aesthetic outcomes. Defects in the craniofacial region are particularly difficult to restore due to the complexity of the tissue structure, geometrical configuration and functional requirements^2^. As it stands, autograft bone substitute is benchmarked as the current gold standard treatment, however, downsides of the harvesting procedure, donor site morbidity and the risk of infection has raised the demand for introducing alternative techniques^3^. This has led to the emergence of novel bone tissue engineering strategies, combining material science, principles of engineering and biology to restore, replace or improve biological function^4^. The successful translation of such systems is the highly dependent presence of a suitable polymeric scaffold to mimic the complex hierarchical structure of bone that supports its diverse mechanical, biological and chemical functions. Polymeric based scaffolds in the field of tissue engineering are divided into synthetic and naturally occurring. Collagen, gelatin and hyaluronic acid are natural polymers that have been approved by the FDA; however, unpredictability of the degradation kinetics causing swelling and sudden bulk degradation and poor mechanical properties has led to emergence of synthetic polymers that are biocompatible and biodegradable^5^. Poly (glycolic acid) (PGA), polylactic acid (PLA) and copolymer (PLGA) are widely used in the clinical environment. However, when large PLGA prosthetics are implanted, a decrease in molecular weight and loss of strength will lead to bulk degradation, releasing high levels of lactic acid and glycolic acid resulting in pH drop and eventually tissue loss^6^. Poly (ε-caprolactone) (PCL) has also been extensively investigated for use in craniofacial reconstruction, however, PCL is slow to degrade^7^ and when implanted often exhibits inadequate cell attachment and insufficient tissue integration due to its hydrophobic nature^8^. Calcium phosphate ceramics are a class of tunable bioactive materials that possess unique properties such as osteoconductivity and osteoinductivity. As a result, they have been used for bone tissue repair and augmentation in parallel with natural/synthetic polymers. They have surface properties that support osteoblast adhesion, proliferation and ultimately promote new bone formation. However, highly reactive/soluble CaPs that often encourage a bioactive behaviour are associated with unpredictability of behaviour in vivo, and fast degradation rate. Therefore, the ratio of CaP incorporation must be carefully designed particularly concerning larger sized defects.

It must be highlighted that aesthetic concerns specifically in the field of maxillofacial reconstruction remain a major challenge as they impose adverse emotional and psychosocial impacts on patients. This calls the need for patient-specific treatments to precisely create custom-fit implants with complex geometries. The utilisation of 3D printing holds great promise in the manufacturing of custom-fit implants in the craniofacial region. Such techniques can allow the production of complex constructs accurately with optimum printing resolution while precise control over the unique contour of the defect is provided. This is a critical factor in designing any biomaterial-based scaffold in the field of tissue engineering as it encourages vascularisation and neo-tissue formation. Ultimately, such constructs can offer a decent alternative to current treatment options to be applied directly without the need for further surgical procedures. Moreover, they can positively impact patients’ quality of life by offering the potential to restore functional outcomes in parallel with aesthetic requirements.

In a recent study, we developed a novel light-curable degradable polymer, referred to as CSMA, targeted at the reconstruction of maxillofacial defects^9^. The main aim was to utilise current tissue engineering approaches to develop a non-cytotoxic polymer combined with calcium phosphate phases, to promote regrowth of damaged maxillofacial tissues. CSMA was synthesised using bis(2-hydroxyethyl) isosorbide (BHIS) as a starting material. The reaction utilises a simple K_2_CO_3_ catalyst which is easily removed. Furthermore, the mechanical properties of the polymers were controlled by the incorporation of triethylene glycol dimethacrylate (TEGDMA), at different ratios. This was employed in conjunction with 3D printing technology to precisely create custom-fit implants with complex geometries. Here, for improved large-scale outcome, higher printing resolution as well as control over the dimension and porosity of the polymer in the absence of CaP fillers, we are investigating the role of CaP addition in vitro and in vivo and the benefits it offers considering long-term rate of bone regeneration.

## Materials and methods

### Polymer synthesis, composite preparation and 3D printing

**Figure 1:**
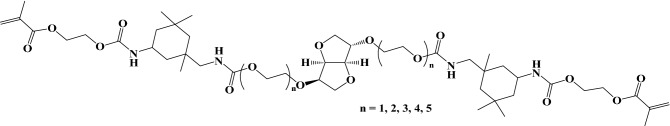
Chemical structure of CSMA.

The monomer, (((((((((((3R,3aR,6S,6aR)- hexahydrofuro[3,2-b]furan-3,6-diyl)bis(oxy))bis(ethane-2,1- 48 diyl))bis(oxy))bis(carbonyl))bis(azanediyl))bis(3,3,5-trimethylcyclohexane-5,1- 49 diyl))bis(azanediyl))bis(carbonyl))bis(oxy))bis(ethane-2,1-diyl) bis(2-methylacrylate) referred to as CSMA-2, shown in Fig. 1., was synthesised as previously explored in a study by Owji et al.^9^ and optimised by Shakouri et al.^10^. To prepare the composite paste, 40, 50 and 60 wt% calcium phosphate filler were mixed with CSMA-2 and 1 wt% CQ using a centrifugal planetary mixer (SpeedMixer, Hauschild Engineering, Hamm, Germany, DAC150.1 FVZ) at 1700RPM for 4 min. The CaP mixture consisted of 10 wt% Mono-Calcium Phosphate Monohydrate (MCPM) with the average size of 53 um and 90% Tri-Calcium Phosphate (TCP) with the average size of 30 um, both purchased form Fluka, UK.

3D Printing was carried out with the aid of Digital Light Processing (DLP) ‘Nobel Superfine’ printer (XYZ Printing, The Netherlands). The synthesised CSMA-2 was combined with 1 weight (wt) % camphorquinone (Sigma-Aldrich, UK). The 3D models were designed via ‘Autodesk AutoCAD 2019’ and prepared and sliced via the XYZ printing software ‘XYZmaker suite’. The model setup for curing was set to 8300 ms with a power intensity of 100 W m^−2^. Once printed, the final models were washed in 99.9% methanol (Sigma-Aldrich, UK) for a total of 5 min to remove any remaining residues.

### Surface remineralisation

#### Raman microscopy

Simulated Body Fluid (SBF) was prepared as per BS ISO 23,317:2014 standard procedure at 37 °C with the final pH of 7 and kept in the fridge. The discs were subsequently immersed in SBF, the chemical changes were recorded up to 28 days of incubation. All spectra were obtained using a Labram spectrometer (Horiba, Jobin Yvon, France), equipped with a 633 nm He–Ne laser, in the range of 850–1700 cm^−1^ and resolution of 2 cm^−1^. For each composite disc, spectra were collected by mapping areas of 40 × 40 μm through a microscope objective (50 ×) and for each area, several spectra were generated and normalised.

#### X-ray diffraction

XRD was used to investigate and identify the precipitation of different calcium phosphate crystals; accordingly, the discs were immersed in SBF and incubated for 28 days. The specimens were then placed flat and XRD spectra were obtained using a Brüker D8 advance diffractometer (Karlsruhe, Germany), with Ni filtered Cu Kα radiation. A Scintillation counter with a step size of 0.02° was finally used for data collection with grazing incidence geometry (incident angle 1°) and a count time of 10 s.

#### SEM imaging

Qualitative visualisation of the composite discs was carried out with the aid of scanning electron microscopy. To image the composite discs, they were coated with 95% gold and 5% palladium (Polaron E5000 Sputter Coater, Quoram Technologies, Laughton, UK) and SEM (Philips XL30 Field Emission SEM, Amsterdam, Netherlands) was used to visualise the surface of the specimen discs.

### Rheology

The viscoelasticity and flow behaviour of the monomer itself (CSMA), and CSMA with CaP were measured using a rheometer HAAKE Viscotester iQ air (Thermo Scientific, UK). A 35 mm Titanium parallel plate geometry was used. A rotational ramp with a shear rate between 0.1 s^−1^ and 100 s^−1^ and a dynamic oscillatory test with steady deformation ɣ = 1.000 at 1 Hz frequency were undertaken for the samples for a step time of 300 s, all test modes were run at room temperature of 25 °C. Collected data were then analysed on the RheoWin software.

### In vitro cytocompatibility Analysis

#### MC3T3 cell culture

MC3T3 cells (Institute of regenerative medicine at the Texas A&M Health Science Centre College of Medicine (USA)) were cultured in minimum essential medium (α-MEM, Gibco BRL) which was supplemented with 10% foetal bovine serum (FBS) (Invitrogen) and 100 U ml^−1^ of penicillin/streptomycin (Sigma-Aldrich, UK). Once the cells reached 80% confluency, they were harvested by trypsin–EDTA (Invitrogen, Paisley, UK) to investigate the proliferation and interaction of the cells with the surface of the specimen discs.

#### Human adipose derived mesenchymal stem cells (ADSCs)

Human ADSCs (Lonza Bioscience (UK) cryopreserved at the primary passage) were cultured in minimum essential medium (α-MEM, Gibco BRL) which was supplemented with 10% foetal bovine serum (FBS) (Invitrogen) and 100 U ml^−1^ of penicillin/streptomycin (Sigma-Aldrich, UK). Once the cells reached 80% confluency, they were harvested by trypsin–EDTA (Invitrogen, Paisley, UK) to investigate the proliferation and interaction of the cells with the surface of the specimen discs (with the seeding density of 1 × 10^4^ cells ml^−1^ where the passage number was 1) followed by addition of a standard (α-MEM, Gibco BRL) and an osteogenic media (Osteogenic Differentiation Basal Medium, Lonza).

#### Metabolic activity

CSMA and CSMA + CaP composite discs were placed in-24 well tissue culture plates (Thermo Fisher Scientific, Loughborough, UK). 10,000 cells per disc were seeded in each well and incubated at 37 °C 5% CO_2_. To determine cell proliferation, at day 1, 7 and 14 and 21 of culture, 100 μl of Alamar Blue dye (Alamar Blue, ABD Serotrec) was added to each well and incubated for a four-hour period. Fluorescence values (excitation wavelength of 530 nm and emission wavelength of 590 nm) were then measured using a Fluroscan Ascent plate reader (Labsystems, Helsinki, Finland).

#### Phalloidin/DAPI Staining

Phalloidin is a bicyclic peptide that allows in-depth qualitative analysis of the cellular cytoskeleton based on the concentration of cytoplasmic actin. Firstly, the discs were stained with phalloidin-iFlour 488 (Abcam, UK) in PBS (1:1000) for 20 min and then fixed with 4.0% paraformaldehyde for 1 h at room temperature. This was followed by staining the cells with 4′, 6-diamidino-2-phenylindole (DAPI) (Abcam, UK) for the duration of 10 min to allow nuclear visualisation. Finally, the discs were washed with PBS and viewed under a fluorescence microscope (Leica – DMIRB).

#### SEM microscopy images for cellular visualisation

Visualisation of the cellular morphology and cell interaction with the material surface was observed via SEM microscopy (Philips XL30 Field Emission SEM, Amsterdam, Netherlands). The discs were initially fixed in 3% glutaraldehyde and 0.1 M cacodylate buffer and stored at 4˚C overnight. This was followed by a series of ethyl alcohol dehydration for 10 min with subsequent drying in hexamethyldisilazane. The specimen discs were finally coated with 95% gold and 5% palladium (Polaron E5000 Sputter Coater, Quorum Technologies, Laughton, UK) for visualisation under the SEM microscope.

#### Quantitative polymerase chain reaction (qPCR) differentiation study

RNA was isolated by the TRIzol (Invitrogen, Paisley, UK) method on days 7, 14, and 21 of culture, and the yield was quantified by a plate reader (Infinite M200, Tecan). cDNA synthesis was carried out using Precision nanoscript 2 reverse transcription kit (Thermofisher, UK) and quantitative PCR was performed with custom-designed and synthesised primers (Thermofisher, UK) for analysis of differentiation pathways of ADSC. Glyceraldehyde 3-phosphate dehydrogenase (GAPDH) was used as the housekeeping gene while Runt-related transcription factor 2 (RUNX2), Collagen type I (COL1A1), Osteocalcin (OCN) and Osteopontin (OPN) were used as genes of interest. The copy number standard curve was performed for each of the primers. These standard curves were used for the absolute quantification of our samples. The results were represented as copy numbers/ul of cDNA.

### Ex ovo chorioallantoic membrane (CAM) assay: angiogenic response

Pro-angiogenic potential of the scaffolds was assessed using an ex ovo CAM assay^11^. Briefly, scaffolds were 3D printed into porous constructs: 6 × 3 mm with 0.5 mm porosity using a DLP technique, sterilised with immersion in methanol, and 15 min UV exposure on each side. Filter paper discs soaked in either PBS (negative) or 10 ng/ml of vascular endothelial growth factor (VEGF) solution (positive) were used as controls. Fertile chicken eggs were incubated at 37.5 °C and 35%–45% humidity in an egg incubator. At day 3 post-incubation, the embryos were transferred to a shell-less culture system with 75%–80% humidity and 37.5 °C incubation temperature. At embryonic day (ED) 10, scaffolds were applied onto the developing CAMs and incubated. On ED13, scaffolds were excised following cryopreservation and 10% formalin fixation. Angiogenesis was examined in all the scaffolds macroscopically by taking photos using a digital camera. ImageJ software was used to analyse the macroscopic photos and calculate the vascular density and the number of bifurcation points for each scaffold.

### In vivo biocompatibility

#### Sample preparation and implantation

**Figure 2:**
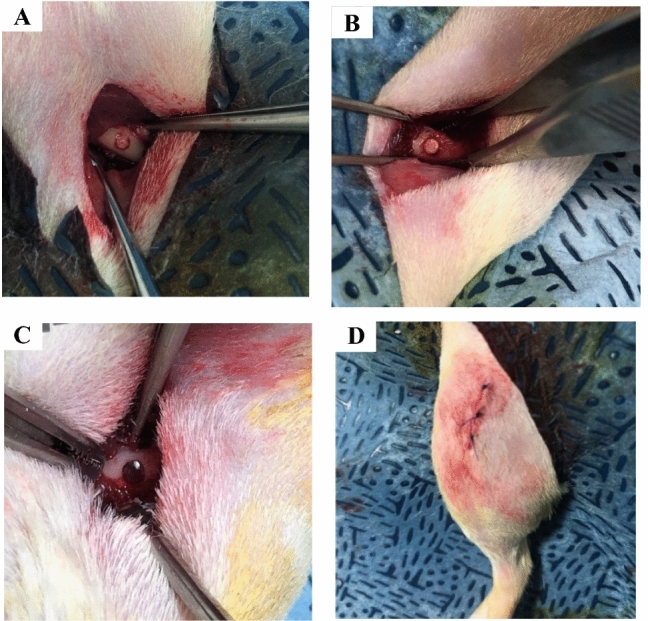
The above images illustrate surgical procedures during the implantation of CSMA and CSMA + 50% CaP in a rat’s femur. The implant was initially inserted into a rat’s femur where the positioning can be observed in a series of images (**A**–**C**). Image D, however, shows the final suturing before retrieval of the specimens after 4 and 8 weeks.

CSMA-2 and CaP incorporated composite (50%) were fabricated with dimensions of 2 mm in diameter and 4 mm in length and photo-polymerised and UV sterilised for 24 h prior to anatomical implantation. The sign of bone regeneration was evaluated in vivo in 10-week-old male Sprague–Dawley rats where a fixed temperature of 20–24 °C with 30–70% humidity was maintained with 12-h light and dark cycles. The rats were shaved and prepared for implant insertion by performing a thorough diiodine wash. CSMA and CSMA + CaP implants (*n* = 3) were then inserted inside the left and right femurs to avoid at depth to avoid any unnecessary movement, shown in Fig. 2. For the aim of histological and micro-CT examination, the rats were sacrificed 4- and 8-week post-implantation, this was in line with the Institutional Animal Care and Use Committee (IACUC) in Dankook University. Post sacrifice of the animals, the tissues were harvested and fixed in a neutral buffer overnight. Serial decalcification and dehydration were subsequently carried out after micro-CT by immersion of the tissues in RegularCal solution (BBC Chemicals, USA) and graded ethanol (20–100% ethanol) and respectively. Finally, the tissue sections were embedded in paraffin wax for histological sectioning and analysis.

#### Micro CT

In order to capture CT images of the harvested samples, the tissue sections were wrapped in a transparent plastic film. Afterwards, the specimens were mounted on a micro-CT (Skyscan 1176, Skyscan, Belgium) for overnight scanning at submicron-level resolution. The voltage of the device was set at 45 kV, the source current of 556 μA was applied through a 0.2 mm aluminium filter and the degree of rotation was arranged at 0.5 degrees with a 180 ms exposure time. Following the capture of the scans, Skyscan NRecon software (Bruker micro-CT, Belgium) facilitated the 3D reconstruction of the images. Finally, to evaluate the volumetric quantification of the new bone, the reconstructed dataset was rendered and segmented.

##### CT quantification: raw data acquisition, processing and volumetric analysis

**Figure 3:**
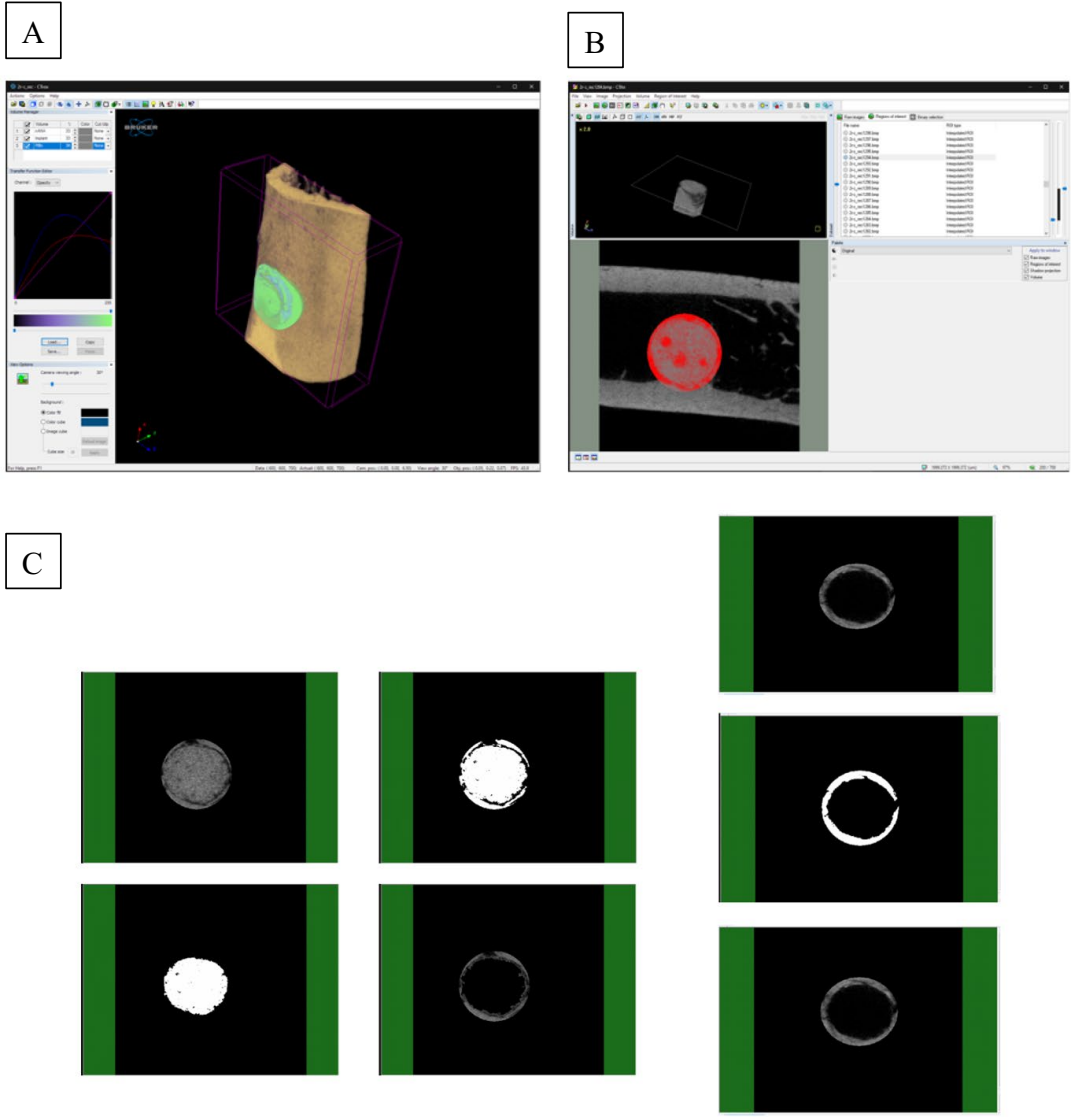
(**A**) represents 3D visualisation of the data on CTVox software, (**B**) is an overview of CTAn software where the Raw Tomographic image sequence is reconstructed based on a multi-dimensional projection into a grayscale image sequence. The bottom left images show the suitable threshold range for segmenting the implant from bone tissue. (**C**) illustrates the region of interest (ROI) loading into the Batch manager (BatMan) for volumetric analysis of the regenerated bone and subsequent sub-data generation for 3D rendering. Data sets are binarised using the range of 50–255 (representing bone) and volumetric analysis was conducted to quantify the amount of bone within the specified region. A subset is generated for rendering the regenerated bone (RBn) and the void space (VOID, 0–49).

Tissue samples were scanned to obtain Raw Tomographic image sequences (Fig. 3). This was followed by Raw Data Processing and Rotation Using NRecon software. The Raw Tomographic image sequence was reconstructed based on a multi-dimensional projection into a grayscale image sequence. The reconstructed data was contained in a separate subfolder < _RECON > and then loaded into the DataViewer software for rotation and data subset preparation. All rotated data sets were cropped to uniform dimensions and generated inside a subfolder < _ROT > . Finally, Volumetric Analysis and 3D Rendering was carried out. The rotated data set was loaded into CTAn software for examination of histogram and thresholding values, defining regions of interest (ROI), programming task list, running 3D/volumetric analysis and generating subsets of binarised image sequence for 3D rendering. Data subsets were similarly loaded into Ctvox software for visualising the implant in 3D space, visualising the new bone tissue surrounding the implant in 3D space and visualising the whole implant site in 3D space.

#### Histology

##### Haematoxylin and Eosin (H&E) + Masson’s trichrome staining

The harvested bone tissue samples were fixed by 4% paraformaldehyde solution for 24 h and then decalcified by RegularCal solution (BBC Chemicals, USA). The decalcified samples were then dehydrated by a graded series of ethanol and embedded in paraffin blocks. The serial paraffin sections cut to 5 μm thickness were deparaffinised and stained by haematoxylin & eosin (HE) and Masson's trichrome (MT) stains. Images of the sections were acquired using a light microscope (Olympus BX50) and analysed using Image-Pro Plus (Media Cybernetics, USA) and the images were processed using ImageJ.

##### Osteocalcin (OCN), osteopontin (OPN) and CD31

The harvested tissue sections were embedded in paraffin wax for histological sectioning and analysis; the serial paraffin sections, cut to 5 μm thickness, were deparaffinized and stained by OCN, OPN and CD31 A light microscope (Olympus BX50) was used to capture images of the sections. For further analysis and processing, Image-Pro Plus (Media Cybernetics, USA) and ImageJ were used respectively.

#### CD31 immunostaining

For immunohistochemical staining, the deparaffinized sections were washed with PBS and incubated overnight at 4 °C with CD31 (Santacruz, USA) antibody. After rinsing with PBS, the sections were incubated in AlexaFluor488-conjugated secondary antibody (Cell Signalling, USA) in a humidified chamber for 1 h at room temperature. The nucleus was stained with DAPI. The fluorescence images were observed and analysed by confocal laser scanning microscope, Zeiss LSM 510 (Carl Zeiss, Germany).

### Statistical analysis

All experiments have three repeats (n = 3) and are presented in the form of mean ± standard deviation. The results were statistically analysed using one-way analysis of variance (ANOVA) at a 95% confidence interval with Tukey’s post hoc test; p < 0.05 was considered to be statistically significant.

### Ethics statement

This study is reported in accordance with ARRIVE guidelines. The animal study was reviewed and approved by the Dankook University Ethics committee DKU-18–032. All experiments were performed in accordance with relevant guidelines and regulations.

## Results

### Surface remineralisation

**Figure 4:**
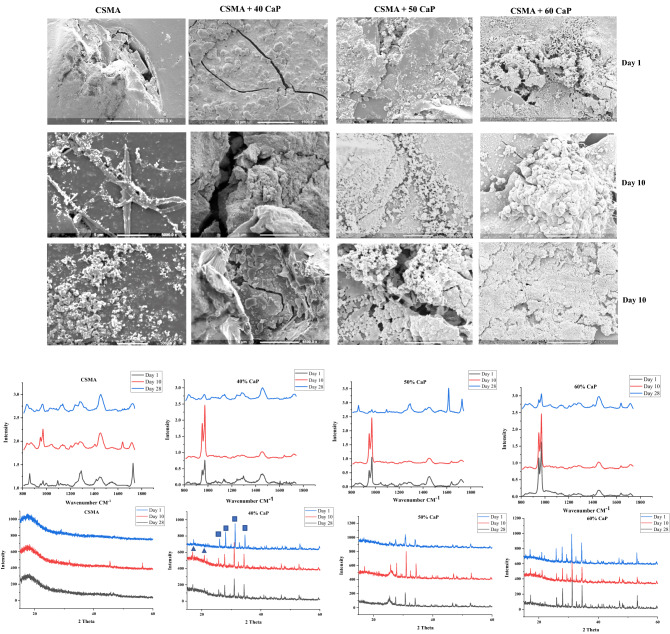
SEM present a series of images of CaP deposition on the surface of the composite discs compared to CSMA. 60% CaP formulation displays the highest quantity of precipitants on the surface, followed by 50 and 40% formulations respectively. Raman microscopy on the first bottom row shows the highest peak at 50% formulation followed by 60 and 40. Subsequently, the presence of CaP precipitates observed via SEM microscopy was confirmed through XRD analysis 1, 10, and 28-days post SBF immersion and incubation.

Initially, surface remineralisation and the potential of Hydroxyapatite (HA) precipitation as a function CaP incorporation in CSMA, at 40, 50 and 60%, was investigated compared to CSMA via SEM, Raman and XRD (Fig. 4). SEM assesses surface remineralisation of CaP incorporated composite discs qualitatively post-incubation in SBF at days 1, 10 and 28. Deposition of CaP particles can be observed on the surface of the discs as a function of time in a series of magnifications. Later time points exhibit a higher quantity of HA-like precipitation; this can be particularly noticed in 50 and 60% CaP formulations. Raman microscopy investigated the amount and type of calcium phosphate deposition on the surface of composite discs post-incubation; the presence of TCP peak at 963 can be observed at all formulations—which is a clear indication of the slow degrading behaviour of tricalcium phosphate—particularly at a higher level in 60% CaP. The highly reactive nature of MCPM has concurrently encouraged above observations where this precipitate is completely washed out from the surface of the discs. Moreover, the drop in the intensity of TCP at day 28 compared to day 10 may suggest the formation of brushite crystals. Finally, an XRD analysis of CaP precipitation in the composite discs is presented. While in Raman microscopy, differences between phases are essentially related to the movement of the main Raman band, XRD allows the identification of different phases as a result of the ongoing reactions inside composites. As the data was collected in θ-θ geometry, the X0rays penetrate a significant distance into the sample bulk and this diffraction technique precisely examines the formation of precipitates inside the CaP incorporated samples. The spectra are dominated by the TCP phase (the samples had 10% MCPM and 90% TCP) (Square symbol). The MCPM cannot be seen due to the low amounts in the samples. The reaction of these two phases shows the formation of brushite (triangle symbol). For the 40 and 50% CaP addition samples, the brushite increases and then decreases with time but for the 60% CaP addition samples, the brushite seems more stable with time. The brushite could either dissolve as it is a more soluble phase, or it can be an intermediate prior to formation of apatite, although there is no evidence of apatite formation in the samples. Interestingly, these findings are not consistent with the quantity measurements obtained via Raman microscopy which is only indicative of surface precipitation. The amorphous nature of CSMA can also observed in the XRD graphs below.

**Figure 5:**
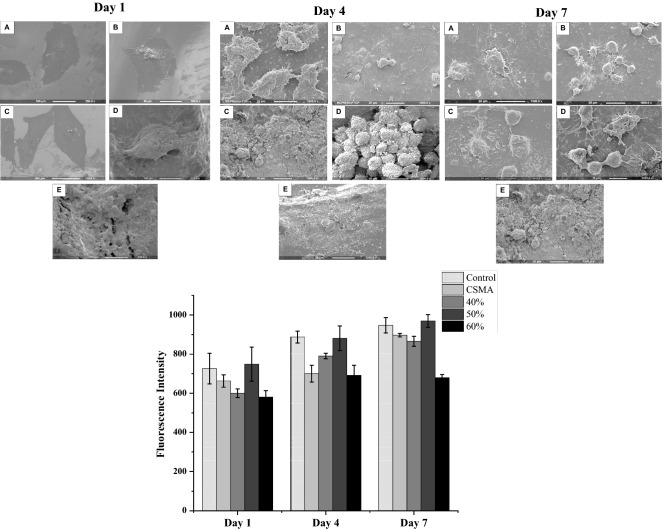
SEM images showing MC3T3 cell proliferation on the surface of control (**A**), CSMA (**B**), 40% CaP (**C**), 50% CaP (**D**) and 60% CAP (**E**) at 1-, 4- and 7-days post-incubation in culture media. Topographical changes can be observed as a function of surface remineralisation, this is particularly apparent at day 7 in D. A uniform layer of cells with clear morphology can also be seen at days 1 and 4 confirming spreading and integration of the cells throughout the surface of the polymer and CaP incorporated discs. Alamar blue metabolic activity of MC3T3 cells on polymer plus 40, 50 and 60% CaP composites, presented on left, shows 50% CaP disc and CSMA did not show any significant difference in the fluorescence intensity value compared to control. The lowest cell proliferation was observed in the 60% CaP disc with exhibiting significantly lower metabolic activity readings (p < 0.05) compared to the remaining discs, this was followed by 40%. The highest statistically significant formulations are presented with an asterisk on top of this graph.

In a previous study cytocompatibility of CSMA was confirmed^9^, here, the incorporation of various filler percentages into the composite and its effect on the metabolic activity of the cells was evaluated. Initially, MC3T3 cells were seeded on the surface of CSMA, 40, 50 and 60% CaP in comparison with a glass coverslip as control; 50% CaP composite disc followed by CSMA did not show any significant difference in metabolic activity compared to control over the 7-day incubation period. The remaining two formulations exhibited a different performance with 60% exhibiting the lowest fluorescence intensity value followed by 40%. SEM microscopy allowed further evaluation of cell proliferation and integration of MC3T3 cells on the surface of CSMA and a range of composite discs. As shown in Fig. 5, the cells can be seen to be adhering and spreading throughout the surface during the incubation period. The morphological changes can also be observed as a function of surface remineralisation. Whereby the clear structure of the cells is apparent at earlier time points, especially day 1- and as more calcium deposition takes place over time, the cells integrate with the material surface. 50% CaP incorporated discs seem to exhibit the highest quantity of cells present at all-time points with no difference with the glass control.

**Figure 6:**
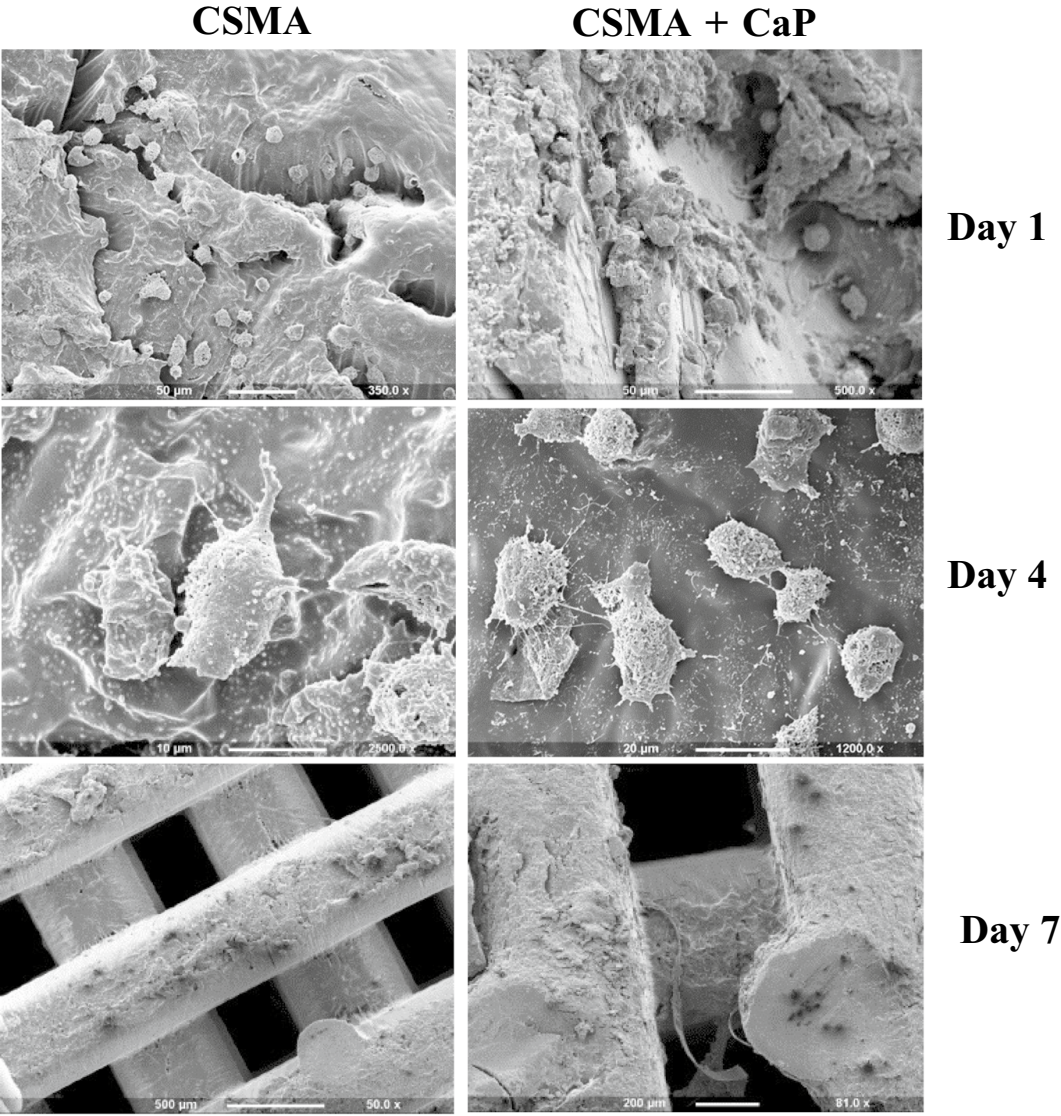
Observation of MC3T3 proliferation on the surface of DLP printed CSMA and CSMA + CaP at 1-, 4- and 7-days post-seeding: the cells exhibit optimum spreading over the incubation period with preference in attachment towards the edges of the samples. Abundant growth of the cells throughout the printed surface can be spotted as early as day 4, where a monolayer of cells is formed. A confluent single cell layer filling the entire surface area is reached at the final time point (day 7).

Following investigating the role of CaP incorporation in CSMA concerning surface remineralisation and biocompatibility, 50% CaP + CSMA and polymer without filler were selected as optimum formulations which will be referred to as CSMA and CSMA + CaP respectively. MC3T3 pre-osteoblasts were then seeded on the surface of the 3D printed scaffolds to assess the attachment and infiltration of these cells onto a 3D printed scaffold, as shown in Fig. 6. The proliferation of this cell line was observed via SEM imaging at day 1-, 4- and 7-days post-incubation culture medium. Interestingly, the attachment of MC3T3 cells can be noticed at day 1 post-incubation, especially at the edge of the polymer matrix. Abundant growth of the cells throughout the printed surface was seen on day 4, where a monolayer of cells was formed. Lastly, on day 7, the cells had reached confluency on the surface of the printed polymer as well polymer with CaP fillers.

**Figure 7:**
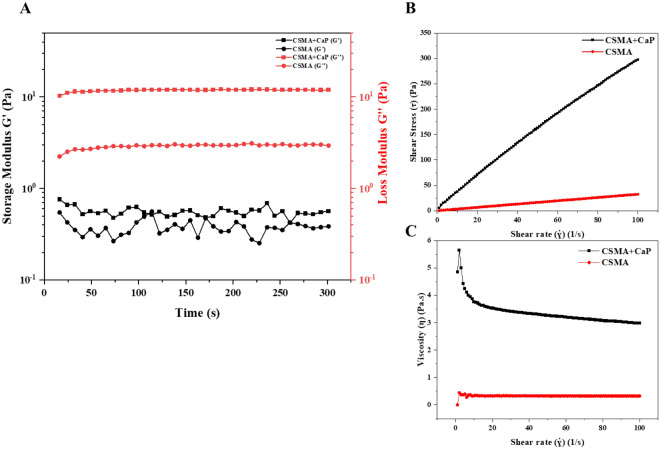
Rheological analyses of CSMA and CSMA + CaP (**A**) storage and loss modulus analyses (**B**,**C**) flow behaviour of the materials shear rate dependant viscosity and shear stress values.

The rheological behaviour of the polymer was explored as a key parameter in using 3D printing fabrication techniques. Flow behaviours of CSMA have become slightly more pseudoplastic fluid like with the incorporation of CaP to the structure (Fig. 7). For shear rates between 1 and 100 s^−^1 in CSMA + CaP as well as CSMA monomer itself, the flow behaviour follows a Newtonian fluid. Regarding the dynamic flow, both formulations have shown higher loss modulus than storage modulus which reflects that the material has a more viscous rather than an elastic response intrinsically. The addition of CaP to CSMA structure has increased the loss modulus from 2 to 10 Pa as seen in Fig. 7A.

**Figure 8:**
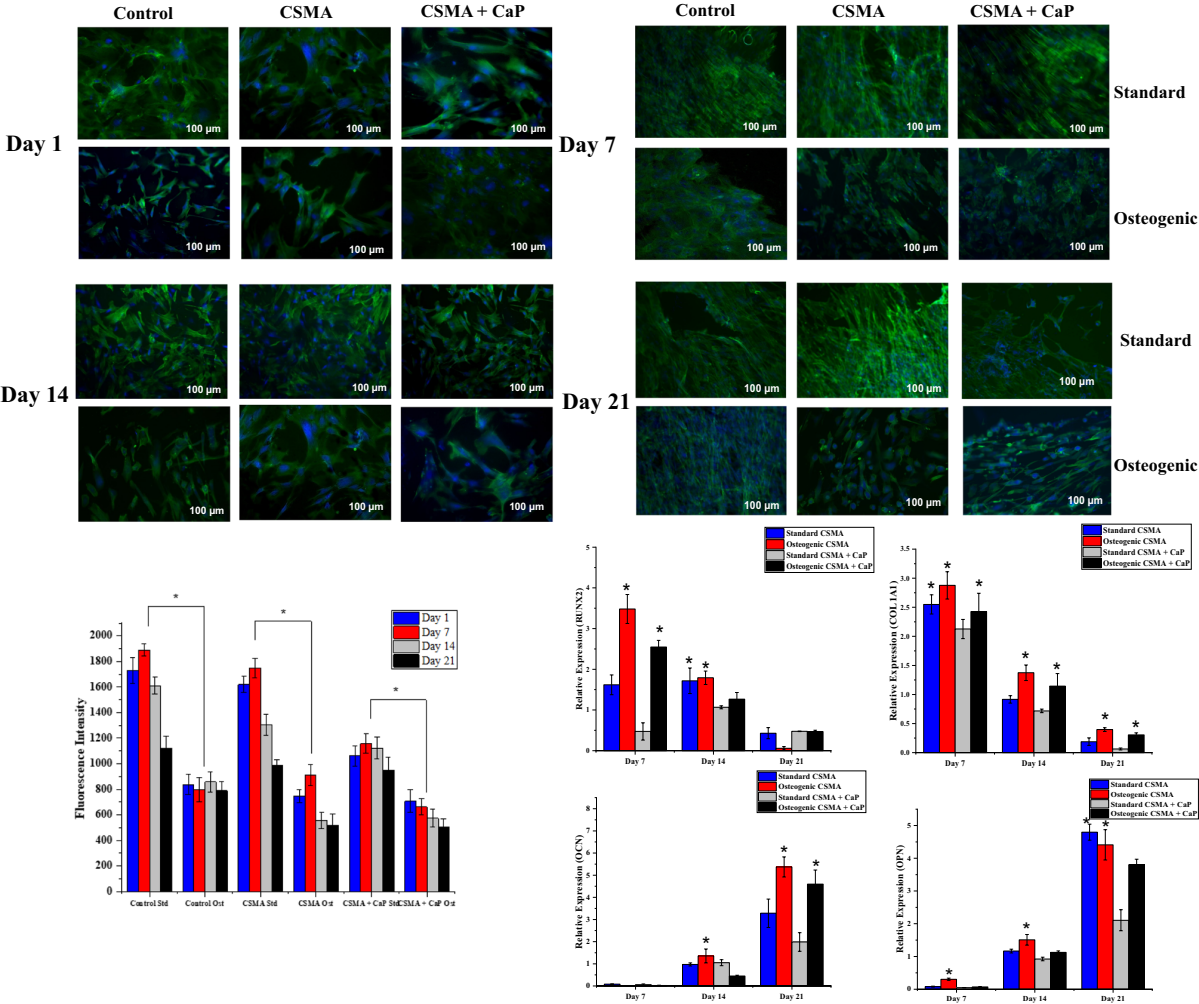
Phalloidin and DAPI staining represent the morphological changes of ADSCs on a coverslip control, CSMA and CSMA + CaP in osteogenic and standard growth conditions over 21 days of culture. The round like morphology at day 14 in the osteogenic sample reveals a sign of osteogenic differentiation, whereas an elongated morphology is observed in the standard condition. The metabolic activity of the cells was evaluated via Alamar blue assay where viability and proliferation of the cells show the highest level of activity at day 1 post culture with a significant difference between the standard and osteogenic media. Finally, osteogenic differentiation markers exhibit upregulation of RUNX2 and COL1A1at day 7, 14 and expression of OCN and OPN at day 21; all formulations show a higher level of relative expression in the presence of osteogenic media. The highest statistically significant values at each time point are presented with an asterisk on top in this graph.

To investigate the presence of primary human ADSCs as well as the morphological changes in 21 days of culture, Phalloidin staining was used to detect cytoskeletal F-actin and DAPI. This allowed analysis of cell interaction with the surface of the polymer comparing an osteogenic environment with the standard media. Figure 8. shows the proliferation of the cells where there is a clear morphological difference between the osteogenic and standard conditions. This is particularly apparent at days 14 and 21, where the cells present a spindle-like and elongated shape in standard media, in contrast to round like morphology indicating an early sign of osteogenic differentiation. Alamar blue fluorescence intensity demonstrates continual viability and proliferation of the cells on all discs. However, a clear difference between the osteogenic and standard media can be observed where the osteogenic condition reveals significantly lower metabolic activity values (P < 0.05) due to a drastic change in pH.

Finally, gene expression of mesenchymal lineage-specific osteogenic differentiation markers including RUNX2, COL1A1, OCN and OPN were studied. ADSCs seeded on tissue culture plastic was used as a control. RUNX2, an early bone marker, and COL1A1, representative of the Extracellular Matrix (ECM) formation, were observed to exhibit the highest level of expression on day 7. Contrary to Alamar blue metabolic activity values, osteogenic condition presented a significantly higher level of gene upregulation in both CSMA and CSMA + CaP. Both OCN and OPN, major non-collagenous proteins involved in the bone matrix organisation, showed higher levels of expression at later time points; the highest being exhibited at 21-day in osteogenic conditions. The overall pattern indicates that CSMA allows a higher level of relative gene expression, particularly in an osteogenic environment compared to CSMA + CaP.

**Figure 9:**
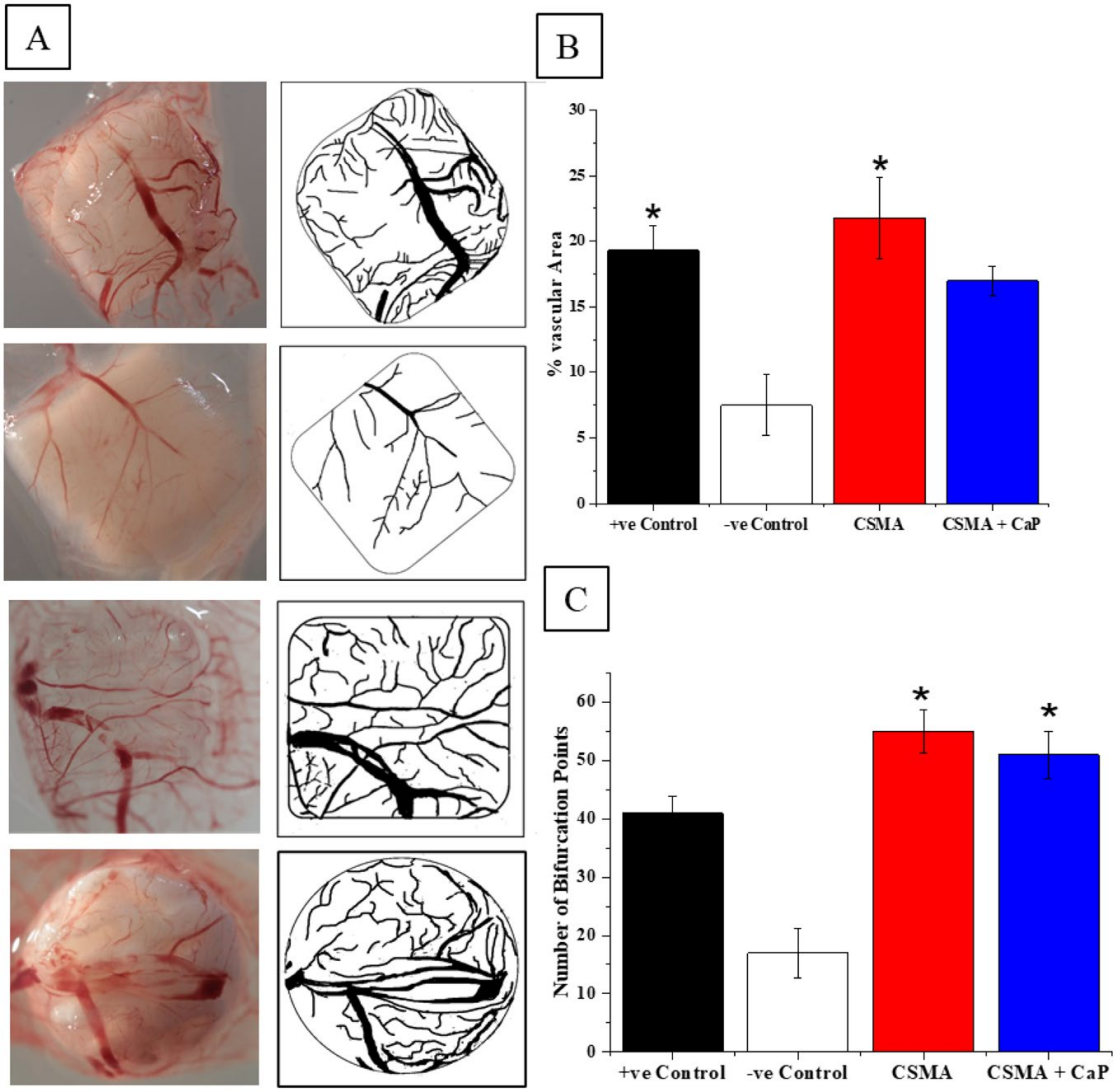
A representative stereomicroscope images of VEGF positive control, PBS negative control, CSMA and CSMA + CaP. B, percentage of vascular area calculated for each sample followed by C, number of bifurcation points, the significantly higher values have been marked with an asterisk *.

The angiogenic response was observed via CAM on ED13 (Fig. 9), and vasculature was studied for each sample: the percentage of the vascular area was the lowest in negative control characterised by a single vessel, with the majority of the area remaining non-vascularised. VEGF positive control presented two large vessels with several capillary plexuses. The vascular invasion for CSMA was the highest among all scaffolds with a cascade of capillaries, they also displayed infiltration of major vessels. CSMA and CSMA + CaP showed the highest number of branching followed by the VEGF positive control.

**Figure 10:**
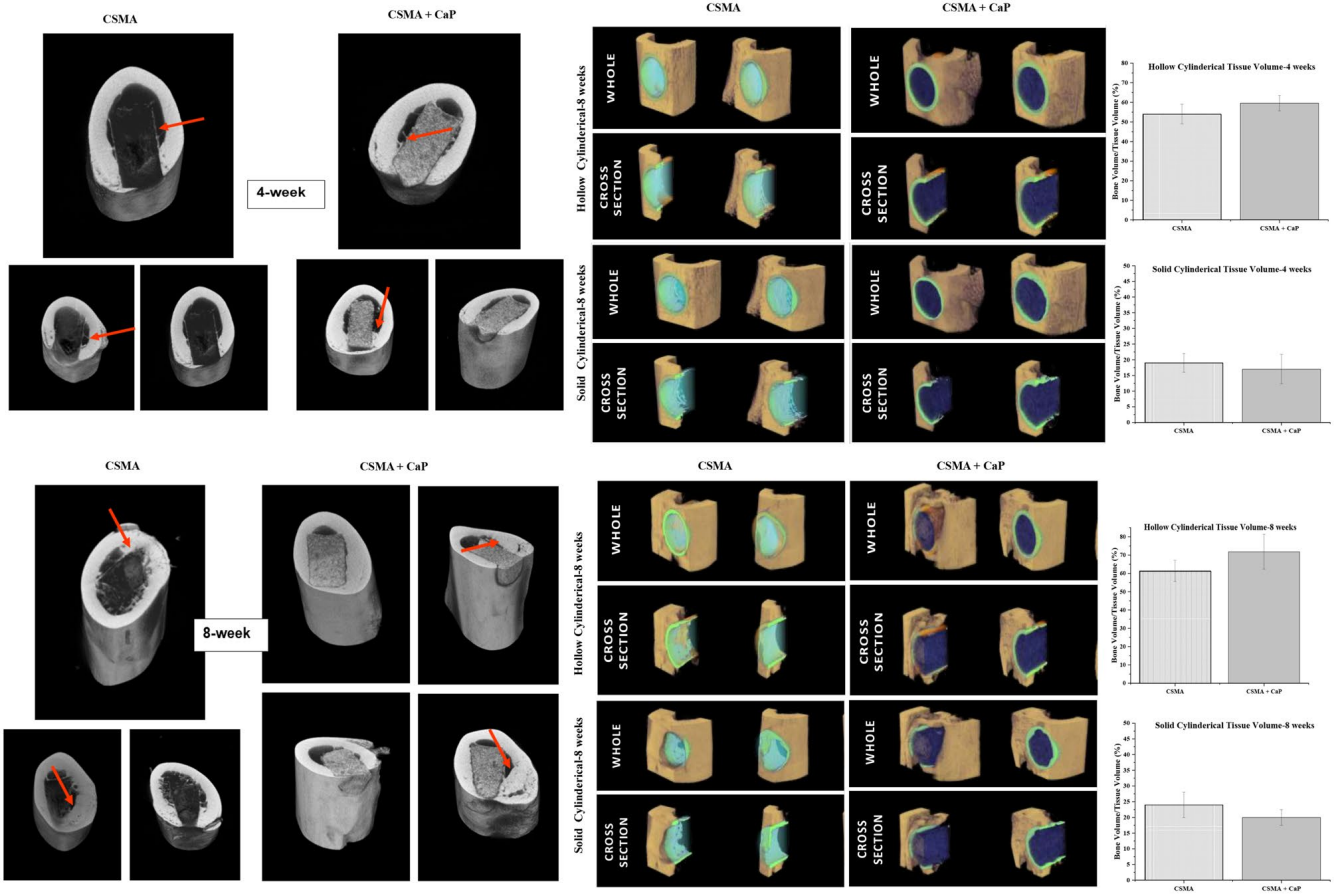
Representative implantation of CSMA polymer and CSMA + CaP composite in the femur of a rat’s model 4 and 8 weeks post-surgical insertion. At week 4, the integration of CSMA based implants with the native bone can be detected. This is particularly obvious where the red arrows are pointing; due to the nature of the CaP filled composite, there is a clearer bone ingrowth in CSMA + CaP. At week 8, an indication of new bone in-growth in both samples can be observed (areas marked with a red arrow). There is also a substantial increase regarding the new bone formation and integration with the native tissue compared to week 4. The bottom schematics present the use of a solid cylindrical as well as a hollow approach in quantitative analysis of new bone formation; CSMA + CaP is shown in yellow while CSMA implant is highlighted in green. Moreover, 3D CT images are also illustrated alongside the graphs for the aim of further qualitative assessment.

The CT images were captured at 4- and 8-weeks post-implantation of CSMA and CSMA + CaP. There is an obvious difference between the two time points as the growth of new bone is clearly present at the latter implanted samples. While signs of osteogenesis can be noticed at CSMA + CaP earlier, there is an ideal integration between the implant and the femur in CSMA samples with no indication of material extrusion. To further evaluate neo-tissue formation at the implant interphase with the native bone, quantification of the collected CT data was carried out via two different approaches; initially, the solid cylindrical tissue volume was calculated which included the volume of the implant plus the growth of new tissues surrounding the material. Secondly, the hollow cylindrical tissue volume was assessed; in this method, the implant volume was subtracted from the image, therefore, the received value was purely representative of the new bone at the integration phase. Figure 10 shows a schematic of these techniques where the implant is highlighted in orange and the new bone is presented in light blue. Quantitative measurements show no significant difference between CSMA and CSMA + CaP. It is also important to highlight the increase in the value of bone formation at 8-week post implantation compared to 4 weeks, from approximately 50% to 70% bone volume/tissue volume ratio.

**Figure 11:**
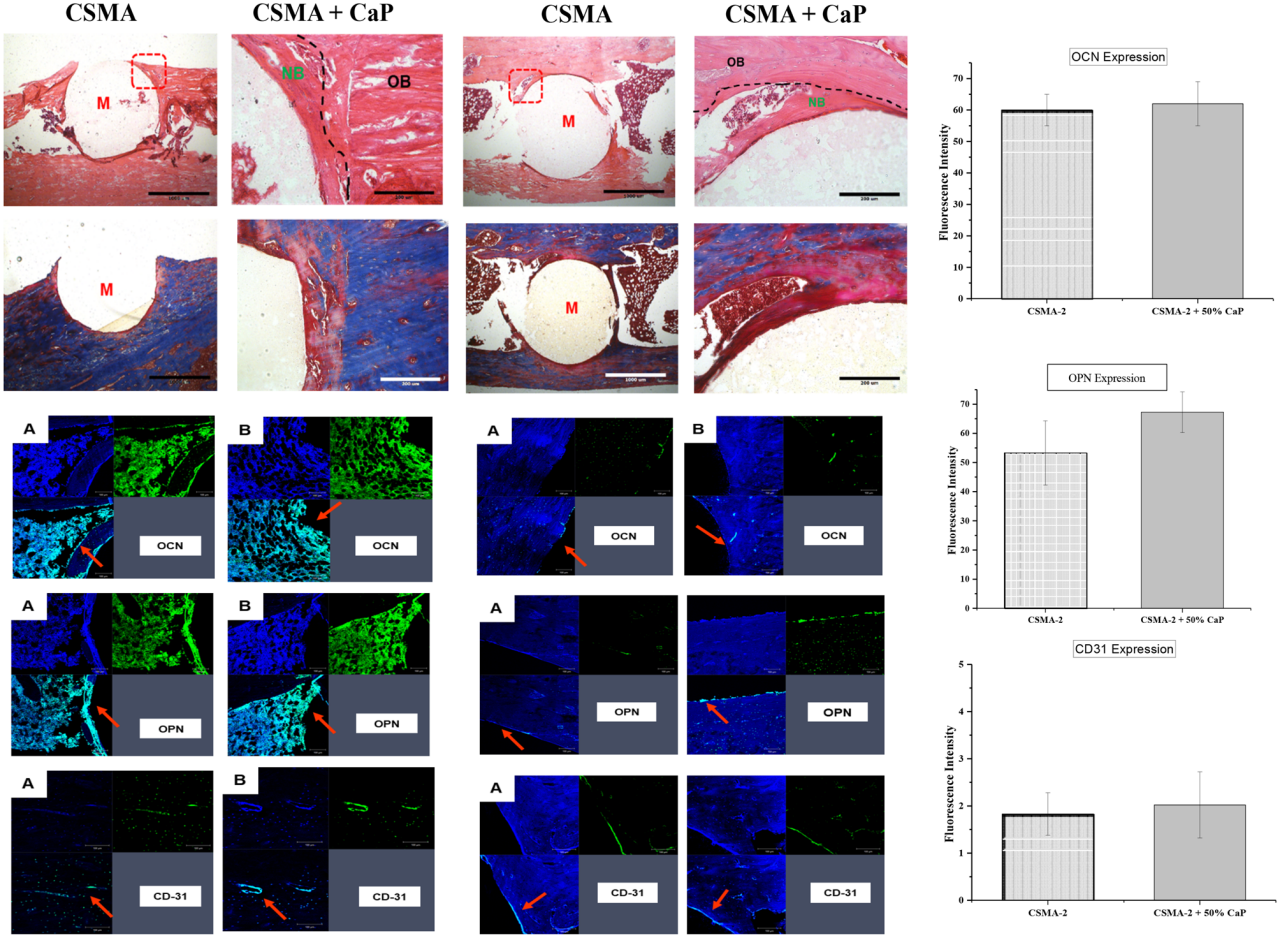
Histological evaluation and illustration of H&E and Masson’s Trichome stains 4- and 8-weeks post-implantation of CMSA and CSMA + CaP: a general overview of the sectioned samples can be observed where a network of collagen fibres is present. In both CSMA and CSMA + CaP implants, cells (white arrow) can be seen around the implant interphase with the native bone. Morphology of the cytoplasmic structures is also visible in both samples. The mineralised bone is presented in blue which contains a cluster of osteoblast-like cells. Bottom images show an assessment of non-collagenous proteins, OPN/OCN and CD31I markers 4- and 8-weeks post-implantation of CSMA (**A**) and CSMA + CaP (**B**): there is a clear difference between 4- and 8-weeks observations in CSMA-based implants where a full network of cells at the border of the native bone, particularly can be observed. Overall, the spreading of the cells is in a uniform pattern across both formulations. Finally, expression of OCN, OPN and CD31 in CSMA and CSMA + CaP was calculated 8 weeks post-implantation: the fluorescence intensity measurements were carried out by ImageJ analysis software to record the intensity within regions of interest (ROIs). Subsequently, a thresholding tool was used to quantify the staining intensity. To compare 3 antibodies across all specimens, staining and image acquisition were performed in parallel for the entire set, where no significant difference can be observed (p > 0.05, n = 3).

To further evaluate the performance of CSMA implants, histological staining was carried out; initially, H&E staining provided a general overview of the sectioned samples where a network of collagen fibres and cells were observed. The presence of cells around the implant interphase with the native bone was also demonstrated; in both CSMA and CSMA + CaP, the morphology of the cytoplasmic structures was clearly visible. As shown in Fig. 11, the implant integrates with the native tissue at 8 weeks post-implantation seems to exhibit a more distinctive colour with less inflammatory cell response compared to the 4-week data. Moreover, an optimum integration can be seen at the margins of the harvested sections. Subsequently, Masson’s trichome allowed identification of neo-bone growth at the implant interface; osteoid presence (shown in red) surrounding the implant verified the process of new bone formation within the 2 months of implantation. The mineralised bone was presented in blue which also contained a cluster of osteoblast-like cells and Haversian canals, particularly at the later time- point, bordering the implant. Subsequently, to detect the presence of non-collagenous bone proteins and signs of vascularisation at the implant interface, osteocalcin and osteopontin and CD31 immunohistochemical staining were performed. In Fig. 11, the presence of viable cells can be seen CSMA and CSMA + CaP with uniform spreading of both DAPI and osteocalcin. The osteopontin and CD31 findings presented a full network of cells at the border of the native bone specifically in CSMA + CaP with a distinct blue colour indicating signs of neo-vascularisation. There seems to be a clear difference at 8 weeks post-implantation compared to the 4-week data in terms of the quantity of the observed cells defined by all three antibodies, OCN/DAPI, OPN/DAPI and CD31. It must be noted that this trend is particularly clear at the margins of CSMA and CSMA + CaP implants. Finally, a quantitative assessment of the viable cells was carried out; no significant difference between CSMA and CSMA + CaP was observed, but a considerable increase in the measurement of cells in all three antibodies was obtained at 8 weeks compared to 4 weeks post-implantation of the samples.

## Discussion

For new bone formation, a successful combination of osteoconduction, osteogenesis and osteoinduction is critical. Therefore, synthetic scaffolds need to incorporate such features to allow restoration of bone defects^12^; this includes suitable bio-functionality, mechanical properties, porosity, biocompatibility and bioresorbability. We have recently introduced a novel photocurable degradable polymer with promising potential for application in maxillofacial reconstruction^9,10^. One of the major benefits of such a system is the rapid photo-polymerisation which is highly advantageous compared to conventional fabrication methods^13^; as a result properties such as fast setting upon light exposure due to rapid cure reaction, improved biological cytocompatibility as a result of reduced emission of volatile organic compounds, low energy requirements, production of in situ scaffolds at room temperature and finally cost-effectivity can be offered^14^. This also opens up a range of processing methods that offer significant advantages in clinical use. In this study, we specifically looked at the potential of this material as a ‘polymer only’ ink for 3D printing applications that can improve resolution and ultimately allow better aesthetic outcomes in patients.

Promoting cellular growth is a fundamental goal in bone tissue engineering^15,16^. The use of calcium phosphates either as pure bioceramics or as cement in combination with polymeric systems have been extensively researched for bone tissue repair and augmentation. These particles possess surface properties that support osteoblast proliferation and play an important role in cell adhesion; they recruit bone marrow stromal cells to damaged sites to induce oseogenesis^17–19^. However, not all types of CaPs have the same biological effect in vivo. There are some concerns regarding the use of calcium phosphate cements such as the difference between the rate of bone regeneration and degradation, the limit of ingrowth due to pore size, lack of mechanical strength^20^ and compromising the printability of a composite system. On the other hand, calcium phosphates with high solubility, which are often sought after due to bioactive properties, can easily change the local pH and ion concentration and adversely affect protein adhesion^21^. Therefore, the type and the amount of CaP addition are two important variables in determining several properties of polymeric based composites. In this study, the filler phase was comprised of 10% MCPM + 90% TCP at 40, 50 and 60 weight percentage to the liquid (CSMA) phase.

With respect to in vitro cell culture, both metabolic activity and visual investigation of the cell morphological changes were assessed; initially, MC3T3 cells were used to investigate the impact of filler addition on the proliferation of the cells. The most optimum formulations were found to be 50% CaP amongst the CaP incorporated composites and CSMA. Suitable mixing between the two phases accounts for the above observation, therefore, the importance of achieving uniform dispersion between the liquid and the powder phase must be considered in hybrid systems^22^. In our study, the addition of 90% TCP and 10% MCPM helped achieve a more uniform distribution of the powder phase into the polymer^23^. Highly bioactive CaP fillers such as MCPM promote in vitro cell proliferation via the formation of nitric oxide which in turn induces bone growth precursor cells for bone tissue regeneration^21^. However, such fillers have been shown to undergo relatively quick dissolution upon water immersion, leading to bulk degradation and volumetric swelling^24^. Albert et al. developed a polylactide based composite where the filler phase was a mixture of TCP and calcium carbonate^25^. Excessive water uptake by tricalcium phosphates resulted in volumetric swelling upon rapid degradation of calcium carbonate fillers. This can also further complicate the potential translation of such systems with defined dimensions in a 3D construct. Further in vitro biocompatibility investigation of CSMA and the impact of CaP addition on upregulation of the genes of interest was carried out. This revealed following ADSC attachment and proliferation, CSMA allows a higher level of gene expression compared to CaP incorporated composites, particularly in an osteogenic environment. Ion release in osteogenic media mimics the bone microenvironment and increases the local concentration of calcium and phosphate ions^26^. This stimulates the formation of bone minerals on the surface and ultimately promotes the expression of osteoblastic differentiation markers such as COL1, OPN, OCN and RUNX2. Even though calcium phosphates have been shown to have a positive impact on osteoblast proliferation and differentiation, in highly viscous hybrid systems, the surface properties are highly dependent on the backbone monomeric phase^27,28^. The presence of cyclohexenes and aliphatic side chains in CSMA facilitates promoting cell differentiation as a result of increasing the surface charge and reducing the permeability barrier of the nuclei^29^; this phenomenon can be compromised in the presence of CaP fillers, particularly if optimum dispersion has not been achieved.

Vascularisation is a vital factor in restoring bone defects^30–32^; this facilitates the development of osteoid deposition and matrix formation within normal bone healing. Essentially, a coupling link exists between the angiogenic and osteogenic cells and signals, which highlights the importance of such interactions. However, in practice, there is a greater need for vascularisation at sites of critical bone defects as they are larger, more complex and require more control in tissue formation and development^33^. CAM ex vivo assessment showed successful anastomosis between implants and the host vasculature. A significant increase in vascular density and number of vessels was particularly observed in CSMA. Recent advancement in 3D printing fabrication techniques has allowed precise control over surface topography, roughness, patterns, and porosity. Roughness topography enhances blood infiltration and controls osteogenic differentiation in vivo^34^; this is via replicating the physical features left by osteoclast activity on the morphology of the bone surface during the process of bone resorption. As a result, scaffolds with tailored surface roughness can direct and continuously support neovascularisation and cell differentiation. Modification of such features as well as higher printing resolution, which has been shown to contribute to enhanced cell proliferation and bone ingrowth is achieved much easier in the absence of CaP fillers^35^. Moreover, as observed in the rheological behaviour of CSMA + CaP, higher resistance to flow is exhibited as a function of shear rate which inevitably impacts the flow and dispersion of the fillers. A viscous polymer only permits a limited amount of CaP incorporation without triggering agglomeration and poor filler dispersion^36^. When a CaP based composite system is implanted in vivo, a great degree of particle dissolution is expected to occur in a dynamic physiological flow; therefore, this can lead to mismatched mechanical properties^37^. Plus, local stress concentrations at the implant interphase can result in reduction in bone mass density due to stress shielding and eventually leads to implant loosening and increases the risk of infection^38^. Therefore, the great performance of CSMA in the absence of CaP can allow tailoring the biodegradability, mechanical strength and various biochemical properties of this scaffold depending on the targeted application.

Finally, in order to assess the physiological performance of the scaffolds and their function in native tissues, in vivo characterisation was carried out; this delivers crucial insights into fundamental mechanisms of biocompatibility in a dynamic microenvironment. Initially, CT images were captured and analysed to detect neo-bone formation. Quantification of micro-CT showed no significant difference between CSMA and CSMA + CaP. Followed by decalcification of the harvested sections, calciphylaxis zones were stained for detecting the presence of collagen around blood vessels in the bone matrix. H&E histological examination showed various structures such as the extracellular matrix, nucleus and cytoplasm and revealed the presence of visible nuclei in all samples. Staining with Masson’s suggested newly formed lamellar bone by demonstrating osteons including Haversian canals in red. Moreover, the presence of blood vessels in a bright pink colour indicated regular bone formation at the border of the native bone at 8 weeks post-implantation. This was followed by further assessment of the implants’ performance by investigating osteocalcin, osteopontin and CD31 antibodies. non-collagenous proteins hold significant importance as critical components of the bone matrix^39^; this is due to intimate interaction with the collagenous matrix as well as mineral components of the bone. Osteopontin is a multifunctional glycoprotein that has been shown to have a high affinity for hydroxyapatite^40^. Within the bone formation process, high levels of pre-osteoblasts, osteoblasts and osteocytes can secrete this glycoprotein in the bone matrix. Similarly, a higher osteocalcin level has been shown to significantly enhance bone mineral density^41^ and correlates with the formation rate of neo-bone. Post histological evaluation of the afore-mentioned antibodies confirmed the presence of osteopontin, osteocalcin and CD31. This was observed at the interphase between CSMA/CSMA + CaP and the native bone with no significant difference in the quantitative measurement.

In conclusion, successful translation of bone tissue engineering strategies to clinical practice is subject to the fabrication of a biocompatible scaffold that closely mimics the natural bone extracellular matrix niche. In this study, extensive in vitro, ex vivo and in vivo characterisation of CSMA has revealed that the performance of this scaffold does not further improve in the presence of calcium phosphates. This outcome allows the fabrication of a polymeric system that is more cost-effective, can offer higher printing resolution as well as control over the dimension and porosity in the absence of CaP fillers. Ultimately, desired aesthetic and functional outcomes will be achieved where small variations in the manufacturing process can easily be adapted to fit specific needs in the replacement of bone defects without requiring additional surgery.

## Supplementary Information

Supplementary Information.

## Data Availability

The datasets generated and/or analysed during the current study are not publicly available due to intellectual property rights (UK patent application number: 1900815.0) but are available from the corresponding author on reasonable request. Gene names and accession numbers can be found in Supplementary information.
