# Supplementary material for: Inclusion of calcium phosphate does not further improve in vitro and in vivo osteogenesis in a novel, highly biocompatible, mechanically stable and 3D printable polymer
Source: Sci Rep. 2022 Oct 10;12:16977. doi: 10.1038/s41598-022-21013-w (PMC9550830; doi:10.1038/s41598-022-21013-w)
Supplement: Supplementary file 1 — Supplementary Information. [file 41598_2022_21013_MOESM1_ESM.docx]

**Suplementary Information**

Figure 12. The above CT images show the femur of a rat’s model at 4-and 8- post creation of a defect where no implant was inserted in the bone post-surgical procedure. The quantification of the empty sample revealed a significantly lower amount of neo-bone formation compared to CSAMA and CSMA + CaP. Histological evaluation of H&E and Masson’s Trichome stains show a disrupted structure at the border with sign of inflammatory response. Finally, OCN (A), OPN (B) and CD31 non-collagenous proteins present a less organised structure where distribution of the cells seems to be far less uniform compared to CSMA and CSMA + CaP.

| **Gene Name** | **Accession number** |
| --- | --- |
| GAPDH | NM_001256799 |
| RUNX2 | NM_001015051 |
| OPN4 | NM_001030015 |
| Col1a1 | NM_007742 |
| OCN | NM_079839 |

Table 1. An overview of gene names and accession numbers used in the differentiation study.
